# AUG-Dependent Translation of Antisense Repeat Transcripts Contributes to Dipeptide Repeat Protein Production in *C9ORF72* Expansion Carriers

**DOI:** 10.3390/cells15181701

**Published:** 2026-09-19

**Authors:** Sandra Almeida, Yuanzheng Gu, Mark W. Kankel

**Affiliations:** 1 Department of Neurology, University of Massachusetts Chan Medical School, Worcester, MA 01605, USA; 2 Neuromuscular & Movement Disorders, Biogen, Cambridge, MA 02142, USA

**Keywords:** dipeptide repeat protein, iPSC-derived neurons, FTD, ALS, ASO, repeat RNA translation

## Abstract

A hexanucleotide repeat expansion in *C9ORF72* is the most common genetic cause of amyotrophic lateral sclerosis (ALS) and frontotemporal dementia (FTD). Bidirectional transcription of the repeat expansion generates sense and antisense RNAs that are translated into dipeptide repeat (DPR) proteins, but the mechanisms of translation initiation remain incompletely understood. Here, we used CRISPR-Cas9 genome editing and steric-blocking antisense oligonucleotides (ASOs) to investigate the role of AUG codons within the antisense repeat RNA. Deletion of an AUG-containing region upstream of the antisense repeats markedly reduced poly(GP) production without affecting antisense RNA levels, demonstrating that this sequence is required for efficient poly(GP) synthesis. We further found that unspliced sense transcripts containing the repeat expansion likely serve as templates for poly(GA) and poly(GR) production in motor neurons. Finally, ASOs targeting the antisense AUG-containing region reduced poly(PR) and poly(GP) levels without altering repeat RNA abundance, supporting a role for AUG-dependent translation of the antisense repeat RNA. These findings provide new insights into the mechanisms of DPR production and suggest that translation-blocking ASOs may represent a therapeutic strategy for *C9ORF72*-associated ALS/FTD.

## 1. Introduction

Amyotrophic lateral sclerosis (ALS) and frontotemporal dementia (FTD) are neurodegenerative disorders frequently caused by a GGGGCC (G_4_C_2_) repeat expansion in the *C9ORF72* gene [1,2], collectively referred to as *C9ORF72* ALS/FTD. The mutation contributes to disease pathogenesis through a combination of loss of normal C9ORF72 protein function and toxic gain-of-function mechanisms. The expanded repeat is bidirectionally transcribed, generating sense and antisense RNA transcripts that can each be translated in all three reading frames to produce dipeptide repeat (DPR) proteins. Sense transcripts generate poly(GA), poly(GR), and poly(GP), whereas antisense transcripts generate poly(GP), poly(PA), and poly(PR). Expanded repeat RNAs are commonly observed as nuclear RNA foci in human autopsy tissues together with DPR proteins, both of which are pathological hallmarks of *C9ORF72* ALS/FTD [3,4,5].

Overexpression studies in cellular and animal models have demonstrated that DPR proteins are highly toxic and disrupt numerous cellular processes [6]. Consequently, considerable effort has focused on identifying the mechanisms that govern DPR production to facilitate the development of therapeutic strategies aimed at preventing their synthesis [7,8,9,10,11]. Despite intensive study, however, the mechanisms through which DPR proteins are translated from *C9ORF72* repeat expansion transcripts in disease-relevant cells remain incompletely understood.

Multiple translation mechanisms likely contribute to DPR production, depending on factors including sequence context, cell type, and level of cellular stress. In particular, translation initiation codons positioned upstream of the repeat expansion may serve as key cis-acting elements that govern DPR synthesis, analogous to the mechanism reported for *FMR1* RNA [12,13]. In *C9ORF72* sense transcripts, a CUG near-cognate initiation codon located 24 nucleotides upstream of the G_4_C_2_ repeat in the poly(GA) reading frame is required for poly(GA) synthesis in reporter constructs [14,15,16,17]. The importance of this CUG codon was subsequently confirmed in the endogenous context of human motor neurons (MNs) carrying more than 1000 G_4_C_2_ repeats, where deletion of an intronic region containing the initiation site abolished poly(GA) production [18]. This effect was selective, as levels of the other sense-derived DPR proteins, poly(GP) and poly(GR), were unaffected. Although the repeat expansion itself could potentially influence ribosome positioning within the intron, ribosome profiling studies demonstrated that removal of the repeat expansion does not eliminate ribosome footprints at the CUG codon used to initiate poly(GA) translation [19]. Collectively, these findings demonstrate that poly(GA) production depends on sequences upstream of the repeat expansion. Poly(GR) translation appears to follow a similar near-cognate initiation mechanism, as deletion of 111 nucleotides upstream of the G_4_C_2_ repeats markedly reduced translation in the poly(GR) frame, whereas deletion of the repeats themselves had little effect in luciferase reporter assays [20].

The sequence context upstream of the repeat expansion appears to be particularly important for the production of poly(GP) and poly(PR), as canonical AUG initiation codons are present in the corresponding antisense RNA reading frames [21]. Using a reporter system, one study found that at least one of three AUG codons in the poly(GP) reading frame is required for protein production [14], a finding that was subsequently replicated [22]. Analysis of human autopsy tissue from *C9ORF72* expansion carriers further suggested that poly(GP) is predominantly generated from antisense transcripts [23]. However, antisense oligonucleotides (ASOs) targeting sense *C9ORF72* RNA substantially reduce poly(GP) levels in expansion carrier-derived cells and cerebrospinal fluid [24,25], indicating that sense transcripts may also contribute to poly(GP) production.

Despite these observations, the contribution of the antisense RNA region containing AUG initiation codons to endogenous poly(GP) and poly(PR) production has not been fully elucidated. Given the prominent role of poly(PR) in *C9ORF72*-associated neurodegeneration [26,27,28,29] and the need to further define the molecular mechanisms underlying its synthesis [15,30], we systematically investigated the function of this region in regulating poly(PR) and poly(GP) expression in MNs and lymphoblastoid cell lines (LCLs) derived from *C9ORF72* hexanucleotide repeat expansion (HRE) carriers. To this end, we employed complementary CRISPR-Cas9 genome-editing and steric-blocking ASO approaches to identify the cis-acting RNA elements required for endogenous DPR synthesis and to assess their potential as therapeutic targets for the selective suppression of toxic DPR expression. By delineating the contribution of antisense RNA initiation regions to DPR protein synthesis, this study advances our understanding of *C9ORF72* disease mechanisms and informs the development of therapeutic strategies aimed at mitigating toxic DPR production.

## 2. Materials and Methods

### 2.1. Generation of C9ORF72 Antisense (AS)-AUGs-Deletion iPSC Lines

The CRISPR-Cas9 system was used to create a deletion in the first intron of *C9ORF72*, 3′ to the G_4_C_2_ repeats at ALSTEM (Richmond, CA, USA). In brief, the Amaxa nucleofector II (program B-016, Lonza, Fremont, CA, USA) was used to transfect iPSCs with two guide RNAs: CGGTGGCGAGTGGGTGAGTG and ATTGCCTGCATCCGGGCCCC. Nucleofected cells were treated with puromycin, dissociated into single cells, placed in 96-well plates, cultured for 14 days, and expanded. Genomic DNA from each clone was extracted with a genomic extraction kit (Zymo Research, Irvine, CA, USA). Clones with the desired homozygous deletion were identified with a PCR amplification assay, and the PCR products were sent for sequencing. At least two iPSC lines containing the expected deletion from each *C9ORF72* expansion carrier were expanded and collected to isolate genomic DNA. The region of interest was amplified by PCR, and the products were sent for sequencing to confirm the identity of each clone.

### 2.2. Motor Neuron Cultures

Motor neurons were differentiated from iPSCs as described [18]. Briefly, iPSC colonies were seeded on Matrigel-coated wells in mTeSR1 medium (StemCell Technologies, Cambridge, MA, USA); 24 h later, the medium was changed to neuroepithelial progenitor (NEP) medium consisting of 1:1 DMEM/F12:Neurobasal, 0.5× N2, 0.5× B27, 0.1 mM ascorbic acid (Sigma-Aldrich), 1× Glutamax, 3 μM CHIR99021 (StemCell Technologies), 2 μM DMH1 (StemCell Technologies), and 2 μM SB431542 (Stemgent, Beltsville, MD, USA) and replaced every other day for 6 days. Progenitor colonies were dissociated with Accutase, seeded 1:6 on Matrigel-coated wells, and cultured in NEP medium containing 0.1 μM retinoic acid and 0.5 μM purmorphamine for 6 days; the medium was replaced every other day. Motor neuron progenitors were lifted, cultured in suspension for 6 days in the absence of CHIR99021, DMH1, and SB431542, and dissociated to single cells with Accutase. Cells were seeded on poly-lysine/laminin-coated wells in motor neuron medium (1:1 DMEM/F12:Neurobasal, 0.5× N2, 0.5× B27, 0.1 mM ascorbic acid, 1× Glutamax, 10 ng/mL BDNF, 10 ng/mL GDNF, 1 μg/mL laminin, 0.1 μM compound E, 0.5 μM retinoic acid and 0.1 μM purmorphamine) for up to 4 weeks. This protocol generated a culture with >90% ChAT^+^ neurons. Cultures were prepared from two sets of lines derived from two *C9ORF72* expansion carriers and respective no-repeat isogenic controls. The no-repeat iPSC lines were first reported in Lopez-Gonzalez et al. [31]. All experiments were done with neurons derived from 3 to 4 independent differentiations.

### 2.3. Lymphoblastoid Cell Lines (LCLs) Cultures

LCLs ND10966, ND11836 and ND16183 were obtained from the Coriell NINDS Repository (Camden, NJ, USA). Lines ND10966 and ND11836 were derived from *C9ORF72* repeat expansion carriers and denoted LCL-1 and LCL-2. Line ND16183, derived from a non-expanded ALS patient, was used to set the background for the DPR measurements. All lines were maintained in RPMI-1640 containing 15% fetal bovine serum at 37 °C with 5% CO_2_.

### 2.4. Measurement of Poly(GR) and Poly(GP)

Poly(GR) and poly(GP) were measured with a Meso Scale Discovery (MSD) immunoassay as described [32]. Briefly, neurons were lysed in ice-cold RIPA buffer (Thermo Fisher Scientific, Waltham, MA, USA) containing a cocktail of protease and phosphatase inhibitors (Thermo Fisher Scientific), sonicated on ice at a 20% pulse rate for 15 s and centrifuged at 16,000× *g* for 20 min at 4 °C. The protein content of supernatants was determined with the Bio-Rad Protein assay reagent (Bio-Rad, Waltham, MA, USA). Neurons or iPSC samples were loaded on a 96-well single-spot plate (MSD; Cat. No. L45XA) pre-coated with a custom-made polyclonal rabbit anti-(GR)_8_ or anti-(GP)_8_ antibodies (1 µg/mL, Covance, Princeton, NJ, USA) and tested in duplicate wells. Serial dilutions of recombinant (GR)_8_ or (GP)_8_ peptide in 1% BSA-TBST (Tris-buffered saline (TBS)-Tween20 (0.05%)) were used to prepare the standard curve. The detection antibodies were anti-(GR)_8_ or anti-(GP)_8_ antibodies previously tagged with GOLD SULFO (GOLD SULFO-TAG NHS-Ester Conjugation Pack, Cat. No. R31AA, MSD) at a concentration of 0.5 µg/mL. Response signals from the assay plate were acquired with a QuickPlex SQ120 instrument (MSD, Rockville, MD, USA). For background correction, electrochemiluminescence (ECL) signal values from neuron/iPSC/LCLs samples lacking repeats were subtracted from the corresponding test samples.

### 2.5. Measurement of Poly(PR)

The poly(PR) assay was performed like the above section, using a custom-made, affinity-purified rabbit polyclonal PR antibody raised against a (PR)20 peptide (Covance) and following a protocol provided by MSD. Briefly, 96-well single-spot streptavidin plates (MSD; Cat. No. L45SA-1) were blocked with Blocker A, coated with biotinylated anti-PR antibody at a concentration of 0.5 μg/mL and incubated overnight at 4 °C. The next day, the plates were washed three times with TBST, loaded with samples in duplicate wells, and incubated for 2 h at room temperature on a shaking platform. The dilutions for the standard curve were prepared with recombinant (PR)_20_ as described above. After three washes, SULFO-tag conjugated detection PR antibody was added to the plates (0.5 μg/mL), incubated for 2 h at room temperature on a shaking platform, and washed again. Lastly, the MSD-Read buffer (2×) was added, and the plates were read on the MSD instrument. Background correction was performed against LCL samples lacking repeats, and the ECL values were presented as relative to LCLs treated with control ASO.

### 2.6. Measurement of Poly(GA)

Neuron pellets were thawed on ice in approximately 120 µL of lysis buffer (1× TBS, pH 7.4, 1 mM EDTA, 1% Triton X-100) with a protease inhibitor cocktail (cOmplete, Sigma-Aldrich, Burlington, MA, USA), vortexed, and incubated at 4 °C for 15 min to fully lyse the pellet. Lysed cells were centrifuged at 14,000× *g* for 20 min at 4 °C. Total protein concentration of the remaining supernatant was determined with the BCA protein assay (Thermo Scientific). Poly(GA) content was measured with an MSD sandwich immunoassay. In this assay, the human/murine chimeric form of anti-GA antibody chGA3 is used for capture, and human anti-GA antibody GA4 and a SULFO-tagged anti-human secondary antibody are used for detection. Poly(GA) concentrations were interpolated from the standard curve using 60X-GA expressed in HEK 293 cells and expressed as ng/mg total protein. For background correction, values from neuron samples lacking repeats were subtracted from the corresponding test samples.

### 2.7. Antisense Oligonucleotide (ASO) Treatment

Steric-blocking ASOs were designed by S. Almeida and purchased from Integrated DNA Technologies (IDT, Coralville, IA, USA). A control ASO was also obtained from IDT. All ASOs were fully 2′-O-methoxyethyl (MOE)-modified, endonuclease-resistant, and designed to sterically block the region surrounding the AUG codon in the poly(PR) reading frame (ASOs 1–3) or the AUG codon at position −194 in the poly(GP) reading frame on the AS RNA (ASO-4). The ASO sequences are: ASO-1: GCATCCGGGCCCCGGGCTT; ASO-2: TGGTGGAATTGCCTGCATCCG; ASO-3: TGGAATTGCCTGCATCCGGG; ASO-4: GCTCGACGCATTTTTACTTTC.

LCLs were treated with ASOs as previously described by Gendron et al. [24]. Briefly, LCLs were seeded at 4.5 × 10^6^ cells per T25 flask and treated with 5 μM control ASO or ASOs 1–4. Three and six days later, cells were re-seeded and retreated with 2.5 μM control ASO or ASOs 1–4. Cells were harvested 10 days after the initial seeding.

### 2.8. Gene Expression

Total RNA was extracted with the Qiagen RNeasy kit (Qiagen, Beverly, MA, USA) and treated with DNase I. RNA (1–2 μg) was reverse transcribed into cDNA with random hexamers or *C9ORF72* antisense–specific reverse primer using the High-Capacity cDNA kit (Thermo Fisher Scientific). Quantitative PCR was done with an Applied Biosystems Quant Studio 3 system and SYBR Select Master Mix (Thermo Fisher Scientific, Waltham, MA, USA). Ct values for each sample and gene were normalized to cyclophilin or actin beta. The relative expression of each target gene was determined with the 2^−ΔΔCt^ method. The following primers were used for qPCR:

*C9ORF72* AS–specific RT rev: 5′ cgactggagcacgaggacactgagggacaagggatggggatc 3′;

AS fwd: 5′ ctctcagtacccgaggctc 3′; rev: 5′cgactggagcacgaggacactga 3′;

V1 fwd: 5′gagaatggaagatcagggtca 3′; rev: 5′ gtatctgcttcatccagcttt 3′;

V2 fwd: 5′ cggtggcgagtggatatct 3′; rev: 5′ gcccaaatgtgccttactct 3′;

V3 fwd: 5′ gggtctagcaagagcaggtg 3′; rev: 5′ agcccaaatgtgccttactc 3′;

V1/V3 pre-mRNA fwd: 5′ tcaaacagcgacaagttccg 3′; rev: 5′ aagtagtggggagagagggt 3′;

intron-1 fwd: 5′ ccccactacttgctctcaca 3′; rev: 5′ ctacaggctgcggttgtttc 3′.

To verify each primer set, PCR products were amplified from cDNA using Taq polymerase, separated on a 2% agarose gel, and visualized with SYBR Safe to confirm the expected amplicon size (Supplementary Figure S1).

### 2.9. Statistical Analysis

Statistical analyses were performed using GraphPad Prism 10 (GraphPad Software, version10.6.1). The statistical tests used, error bars displayed, and numbers of biological replicates are indicated in the figure legends. Multiple-comparison corrections were performed using Dunnett’s test. Differences were considered statistically significant at *p* < 0.05.

## 3. Results

### 3.1. Deletion of a Region Containing Multiple AUG Initiation Codons in the Antisense Repeat RNA Greatly Reduces Poly(GP) Protein Levels in C9ORF72 iPSC-Derived Motor Neurons

Poly(GP) is the only DPR protein that can potentially be generated from both the sense and antisense repeat RNAs. In the sense direction, a stop codon located immediately upstream of the G_4_C_2_ repeat expansion and in the poly(GP) reading frame may prevent poly(GP) translation. In contrast, in the antisense direction, three AUG start codons are in-frame with poly(GP). Unlike the sense RNA, the AS RNA lacks an upstream stop codon that would prevent translation into the C_4_G_2_ repeats.

**Figure 1:**
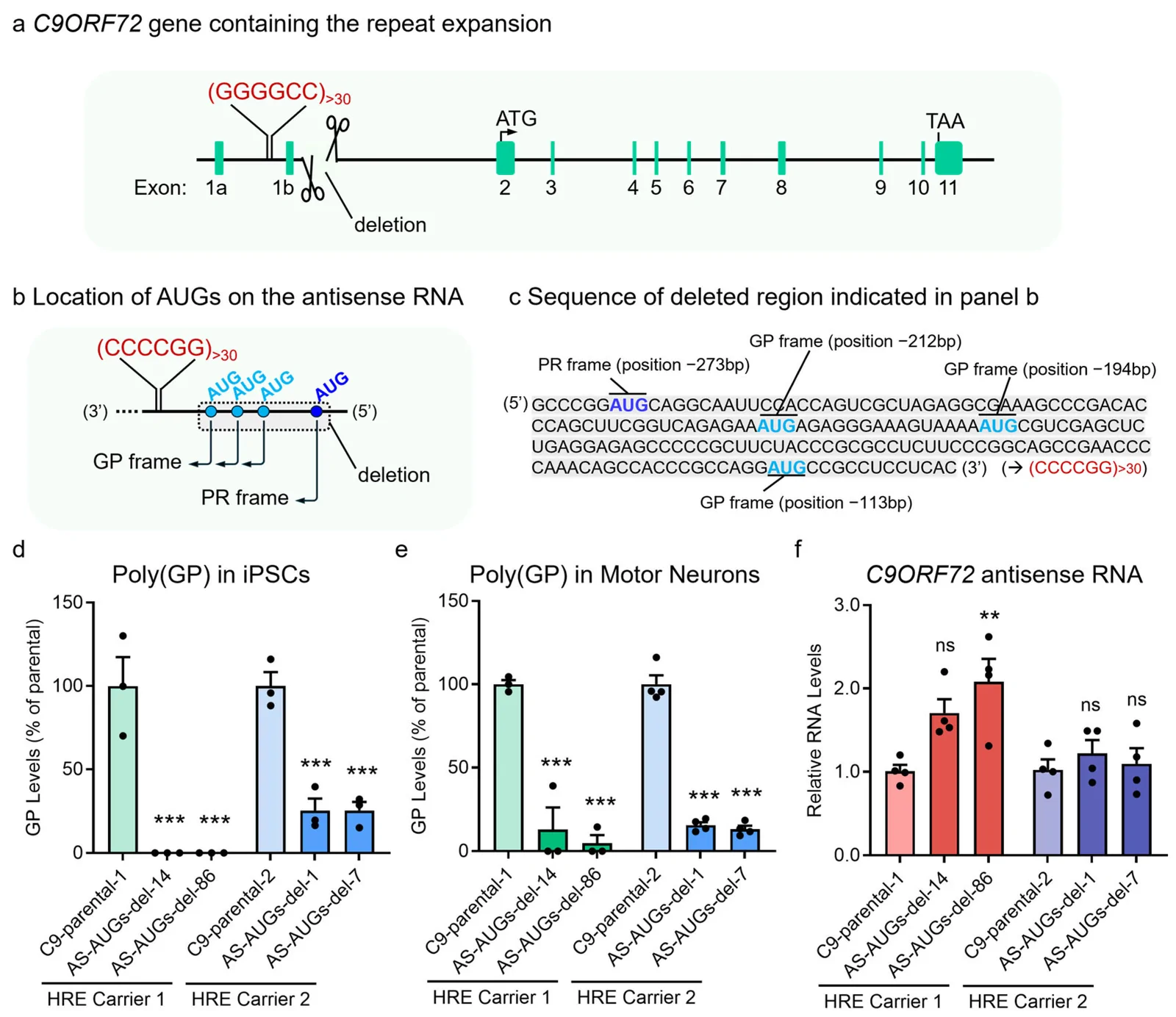
Deletion of the AUG codon-containing region in the antisense repeat RNA reduces poly(GP) protein levels in *C9ORF72* iPSCs and motor neurons. (**a**) Schematic of the *C9ORF72* gene showing the location of the 181 bp deletion generated by CRISPR-Cas9. (**b**) Schematic of the antisense repeat RNA indicating the location of the AUG codons in the poly(GP) and poly(PR) reading frames. (**c**) Nucleotide sequence of the antisense RNA region deleted by CRISPR-Cas9, with the AUG codons in the poly(GP) and poly(PR) reading frames highlighted. (**d**,**e**) Poly(GP) protein levels in parental and antisense AUGs-deletion (AS-AUGs-del) iPSCs (**d**) and motor neurons (**e**) derived from two *C9ORF72* (C9) hexanucleotide repeat expansion (HRE) carriers. (**f**) Relative expression levels of *C9ORF72* antisense RNA in parental and AS-AUGs-del motor neurons, assessed by quantitative PCR. Values represent the mean ± s.e.m. from three to four independent differentiations. ** *p* < 0.01, *** *p* < 0.001 (one-way ANOVA with Dunnett’s multiple-comparisons test); ns, not significant.

To determine whether these AUG codons are required for antisense poly(GP) production, we investigated whether they function as translation initiation sites. Because it was unknown which AUG codon initiates poly(GP) translation, and because mutation of a single AUG could potentially permit initiation from another AUG, we eliminated all three AUG codons simultaneously. Using CRISPR-Cas9 genome editing, we generated a homozygous deletion downstream of the G_4_C_2_ repeat expansion in iPSC lines derived from two *C9ORF72* HRE carriers (AS-AUGs deletion; Figure 1a,b). The deletion removed a 181-nucleotide region containing the three AUG codons in the poly(GP) reading frame and one AUG codon in the poly(PR) reading frame of the AS repeat RNA (Figure 1b,c). Importantly, analysis of the sequence demonstrated that the deletion did not create any alternative initiation codons in the poly(GP) or poly(PR) reading frames within the remaining sequence upstream of the C_4_G_2_ repeats.

We tested multiple guide RNA combinations in an effort to generate smaller deletions positioned closer to the AUG codons. Although the smallest theoretically achievable deletion spanned 163 bp, the region is highly GC-rich, and we were unable to obtain additional edited iPSC lines with smaller deletions. Two AS-AUGs deletion clones from each parental *C9ORF72* iPSC line were selected for further analysis. All four edited iPSC lines contained the same homozygous 181-nucleotide deletion (Figure 1c). We first assessed the impact of this deletion on poly(GP) production using a previously established Meso Scale Discovery (MSD) immunoassay [32]. Soluble poly(GP) levels were nearly undetectable in AS-AUGs deletion iPSCs derived from HRE carrier 1 and were reduced by more than 80% in those derived from HRE carrier 2 compared with their respective parental lines (Figure 1d). Similar reductions in poly(GP) protein levels were observed following differentiation of all iPSC lines into MNs (Figure 1e). The differences observed between the two carrier lines likely reflect intrinsic variability between genetic backgrounds rather than differences in the biological impact of the AS-AUGs deletion. Importantly, AS repeat RNA levels, measured by quantitative RT-PCR, were not decreased in the AS-AUGs deletion lines (Figure 1f). These findings indicate that the reduction in soluble poly(GP) results from impaired translation rather than from a decrease in the abundance of the RNA template.

**Figure 2:**
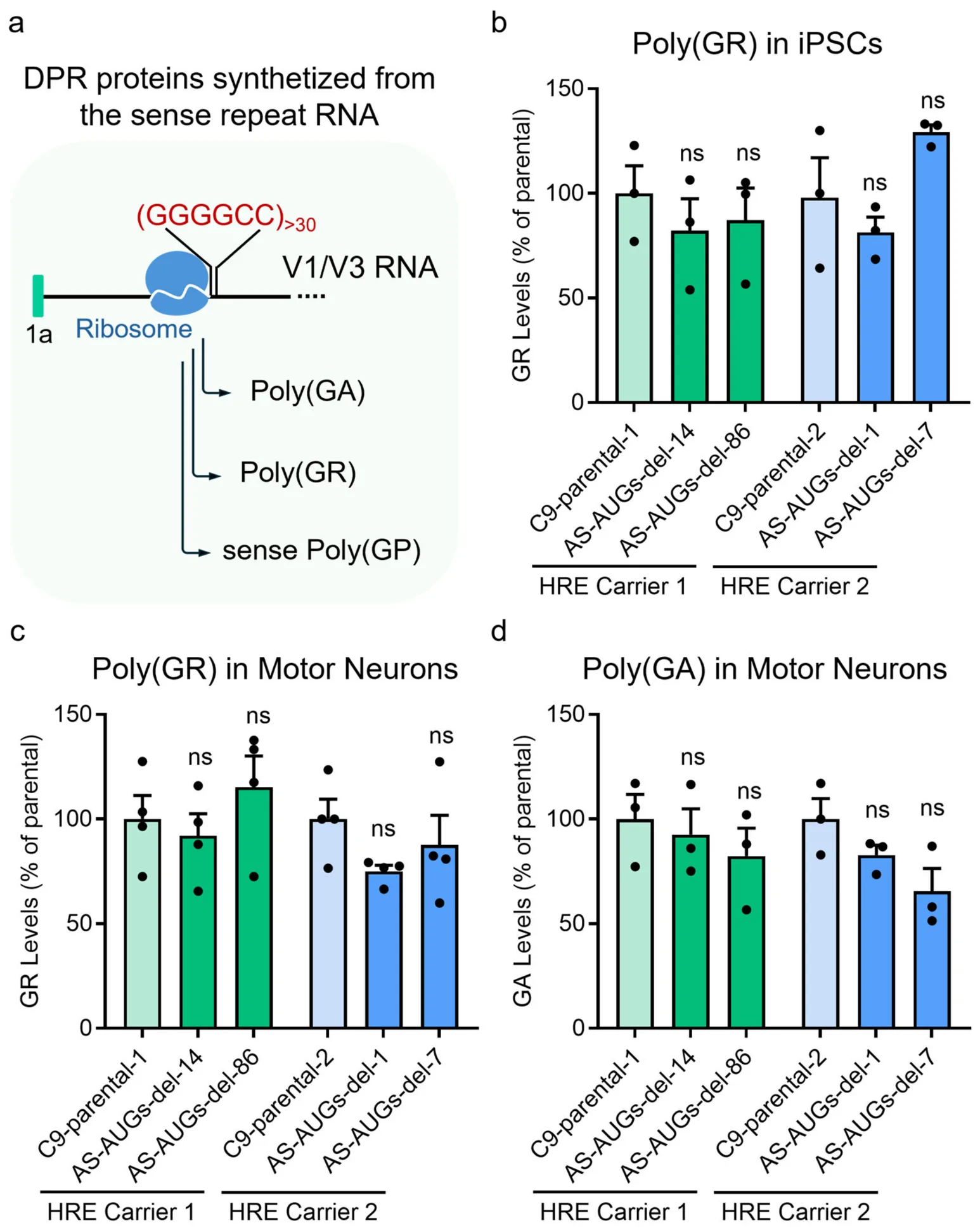
Poly(GR) and poly(GA) protein levels in AS-AUGs-deletion iPSC-derived motor neurons. (**a**) Schematic of the sense repeat RNA (V1/V3 pre-mRNA) indicating the DPR proteins produced from the three possible reading frames. (**b**,**c**) Poly(GR) protein levels in iPSCs (**b**) and motor neurons (**c**), and (**d**) poly(GA) protein levels in motor neurons derived from two *C9ORF72* (C9) hexanucleotide repeat expansion (HRE) carriers, measured by Meso Scale Discovery immunoassays. Values represent the mean ± s.e.m. from three to four independent differentiations; ns, not significant (one-way ANOVA with Dunnett’s multiple-comparisons test).

To determine whether deletion of the AS AUG-containing region affected translation from the sense repeat RNA, we measured levels of the sense-derived DPRs poly(GR) and poly(GA) (Figure 2a). Neither poly(GR) nor poly(GA) levels were altered in AS-AUGs deletion iPSCs or MNs (Figure 2b–d). If the majority of poly(GP) synthesis originated from translation of the sense repeat RNA, poly(GP) levels would be expected to remain largely unchanged following deletion of the AS AUG-containing region. Instead, we observed a marked reduction in poly(GP) protein levels, suggesting its translation derives from the AS RNA.

While these experiments do not distinguish between a direct role of the AUG codons and other cis-regulatory elements contained within the deleted region, they demonstrate that the AUG-containing sequence upstream of the C_4_G_2_ repeats is required for production of the majority of soluble poly(GP). Taken together, these findings indicate that antisense repeat RNA is the predominant source of soluble poly(GP) in *C9ORF72* neurons.

### 3.2. Unspliced C9ORF72 V1/V3 Pre-mRNAs Containing the Repeat Expansion Serve as Templates for Poly(GA) and Poly(GR) Production

Since levels of the sense-derived DPR proteins poly(GA) and poly(GR) were unaffected by the AS-AUGs deletion, unlike poly(GP), we next sought to determine whether any sense *C9ORF72* transcripts were altered by this deletion. We noted that the AS-AUGs-region deletion is located near the end of exon-1b (Figure 1a), the first exon of *C9ORF72* transcript variant 2 (V2), thereby changing the 5′ splice site sequence from GUGAG to GUGAC. This raised the possibility that removal of intron-1 from V2 transcripts might be impaired.

To test this hypothesis, we developed a V2-specific quantitative RT-PCR assay with a forward primer spanning the exon-1b-exon-2 junction, enabling selective detection of properly spliced V2 transcripts. Mature V2 mRNA was readily detected in parental C9 MNs but was undetectable in AS-AUGs-deletion MNs (no Ct values were available for the deletion clone lines), indicating that the deletion impairs efficient removal of intron-1 from V2 transcripts. Because intron-1 of V2 does not contain the repeat expansion, this splicing defect is unlikely to directly influence DPR production.

**Figure 3:**
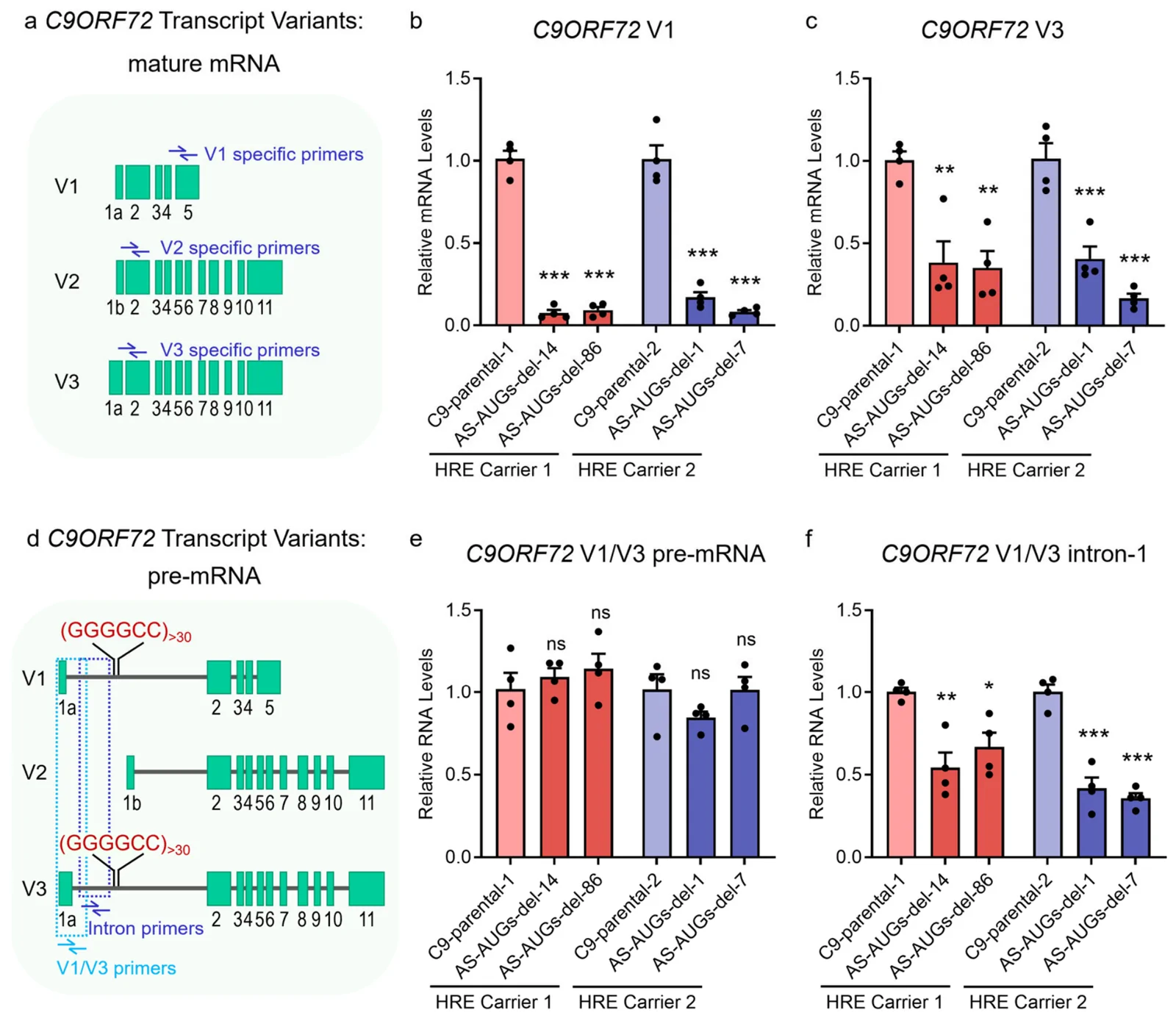
Unspliced *C9ORF72* V1/V3 pre-mRNAs containing the repeat expansion serve as templates for poly(GA) and poly(GR) production. (**a**) Schematic representation of the spliced mRNA transcripts corresponding to *C9ORF72* variants V1, V2, and V3. The locations of the primer sets used to detect each transcript variant are indicated. (**b**,**c**) Relative expression levels of mature *C9ORF72* V1 (**b**) and V3 (**c**) transcripts in parental *C9ORF72* (C9) and AS-AUGs-deletion neurons, assessed by quantitative PCR. (**d**) Schematic representation of the pre-mRNAs corresponding to the three *C9ORF72* transcript variants (V1, V2, and V3). The location of the primer sets used to detect V1/V3 pre-mRNA and intron-1 is indicated. (**e**,**f**) Relative expression levels of *C9ORF72* pre-mRNA (**e**) and intron-1-containing transcripts (**f**) common to both V1 and V3 in parental C9 and AS-AUGs-deletion neurons, assessed by quantitative PCR. Values represent the mean ± s.e.m. from three to four independent differentiations. * *p* < 0.05, ** *p* < 0.01, *** *p* < 0.001 (one-way ANOVA with Dunnett’s multiple-comparisons test); ns, not significant.

We next developed quantitative RT-PCR assays to quantify mature V1 and V3 transcripts, which differ from V2 by incorporating exon-1a rather than exon-1b (Figure 3a). In contrast to V2, properly spliced V1 and V3 transcripts were detected in AS-AUGs-deletion MNs, although their levels were reduced relative to the corresponding parental C9 MNs (Figure 3b,c). The mechanism underlying the reduction in mature V1 and V3 transcripts remains unclear. However, given that poly(GA) and poly(GR) levels were unchanged, these findings suggest that properly spliced V1 and V3 transcripts are unlikely to be necessary for synthesis of these DPRs.

To investigate this possibility further, we examined the effects of the deletion on unspliced transcripts containing intron-1. Because V1 and V3 originate from the same transcription start site and are generated through alternative splicing of a common pre-mRNA, they cannot be distinguished at the pre-mRNA level. We therefore used a primer set that detects transcripts containing both exon-1a and intron-1, enabling quantification of the combined levels of V1 and V3 pre-mRNAs (Figure 3d). In AS-AUGs-deletion MNs, V1/V3 pre-mRNA levels were comparable to those observed in the corresponding parental neurons (Figure 3e), indicating that unspliced sense transcripts containing the repeat expansion are maintained despite the deletion. In contrast, intron-1 RNA levels were reduced by approximately 50% in AS-AUGs-deletion MNs relative to parental controls (Figure 3f). Because the intron-1 assay detects both excised intron RNA and intron-containing transcripts, this decrease most likely reflects reduced abundance of excised intron-1 following impaired splicing.

Taken together, these findings, along with the observation that soluble poly(GA) and poly(GR) levels remain unchanged in AS-AUGs-deletion MNs (Figure 2b–d), support the conclusion that unspliced V1/V3 pre-mRNAs containing the sense repeat expansion serve as the principal templates for poly(GA) and poly(GR) production in human neurons under these experimental conditions.

### 3.3. Poly(PR) Production Depends on an AUG-Containing Sequence Upstream of the Antisense Repeat RNA

**Figure 4:**
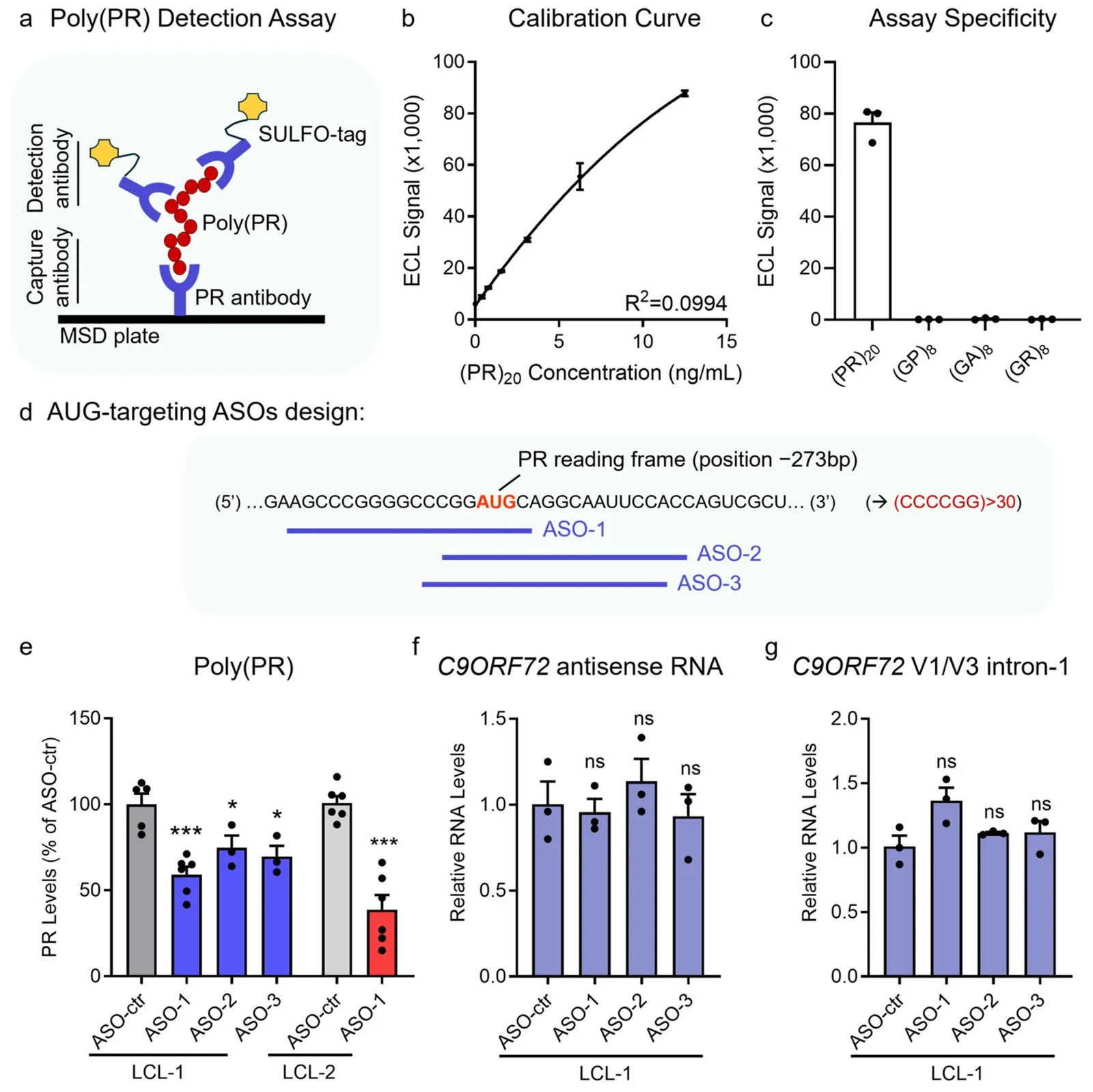
Antisense oligonucleotides (ASOs) sterically blocking an AUG start codon in the antisense repeat RNA suppress poly(PR) protein synthesis. (**a**) Schematic of the Meso Scale Discovery (MSD) assay developed to detect poly(PR) using a custom antibody. (**b**) Validation of the assay using a calibration curve generated with increasing concentrations of a synthetic (PR)_20_ peptide. (**c**) Assay specificity evaluated using (PR)_20_, (GP)_8_, (GA)_8_, and (GR)_8_ peptides, demonstrating selective detection of poly(PR). (**d**) Schematic of steric-blocking ASO design targeting the AUG start codon in the poly(PR) reading frame. (**e**) Poly(PR) protein levels in two *C9ORF72* lymphoblastoid cell lines (LCLs) following treatment with steric-blocking ASOs for 10 days. (**f**,**g**) Relative expression levels of *C9ORF72* antisense RNA and sense V1/V3 intron-1 in *C9ORF72* LCL-1 cells treated with steric-blocking ASOs for 10 days, assessed by quantitative PCR. Values represent the mean ± s.e.m. from three to six independent cultures. * *p* < 0.05, *** *p* < 0.001 (one-way ANOVA with Dunnett’s multiple-comparisons test); ns, not significant.

In addition to eliminating the three AUG start codons in the poly(GP) reading frame, the AS-AUGs deletion also removed the single AUG codon present in the poly(PR) reading frame (Figure 1a–c). We therefore investigated whether this AUG plays an important role in poly(PR) production, analogous to the role of the AUG codons required for efficient poly(GP) synthesis. To address this question, we generated a custom antibody against poly(PR) and developed a highly specific MSD immunoassay that does not cross-react with poly(GP), poly(GA), or poly(GR) (Figure 4a–c).

The assay robustly detected poly(PR) following overexpression in HEK293 cells, with sensitivity comparable to our poly(GR) assay. However, despite its robust performance in overexpression systems, the assay did not reliably detect endogenous poly(PR) in MNs derived from the two *C9ORF72* HRE carriers. Consequently, we were unable to determine whether deletion of the AUG codon affected poly(PR) levels in these neurons.

To assess poly(PR) production in a cell type with higher detectable levels of the protein, we obtained LCLs from two previously characterized *C9ORF72* HRE carriers (ND10966, LCL-1; ND11836, LCL-2) [33]. As an alternative to the CRISPR-Cas9 deletion strategy, we designed steric-blocking ASOs complementary to the region containing the AUG codon in the poly(PR) reading frame (Figure 4d). These ASOs were administered to the two *C9ORF72* LCLs using a 10-day treatment paradigm previously employed to evaluate the effects of *C9ORF72*-targeting ASOs on DPR production [24]. LCL-1 cells were treated with three independent ASOs targeting the AUG-containing sequence (ASOs 1–3). All ASOs reduced poly(PR) protein levels, with reductions of approximately 40%, 25%, and 30% for ASO-1, ASO-2, and ASO-3, respectively (Figure 4e). The effect of ASO-1 was independently validated in LCL-2 cells, in which poly(PR) levels were reduced by more than 60%. The inability of the ASOs to completely abolish poly(PR) production may reflect incomplete target engagement and/or limited accessibility of the target region. Importantly, ASO treatment did not alter antisense repeat RNA levels (Figure 4f) or levels of sense repeat-containing RNA (*C9ORF72* intron-1; Figure 4g), indicating that the reduction in poly(PR) was not driven by changes in transcript abundance. Thus, despite substantial reductions in poly(PR) protein levels, repeat RNA abundance remained unchanged, consistent with the AUG-containing region contributing to translation rather than transcription or RNA stability.

**Figure 5:**
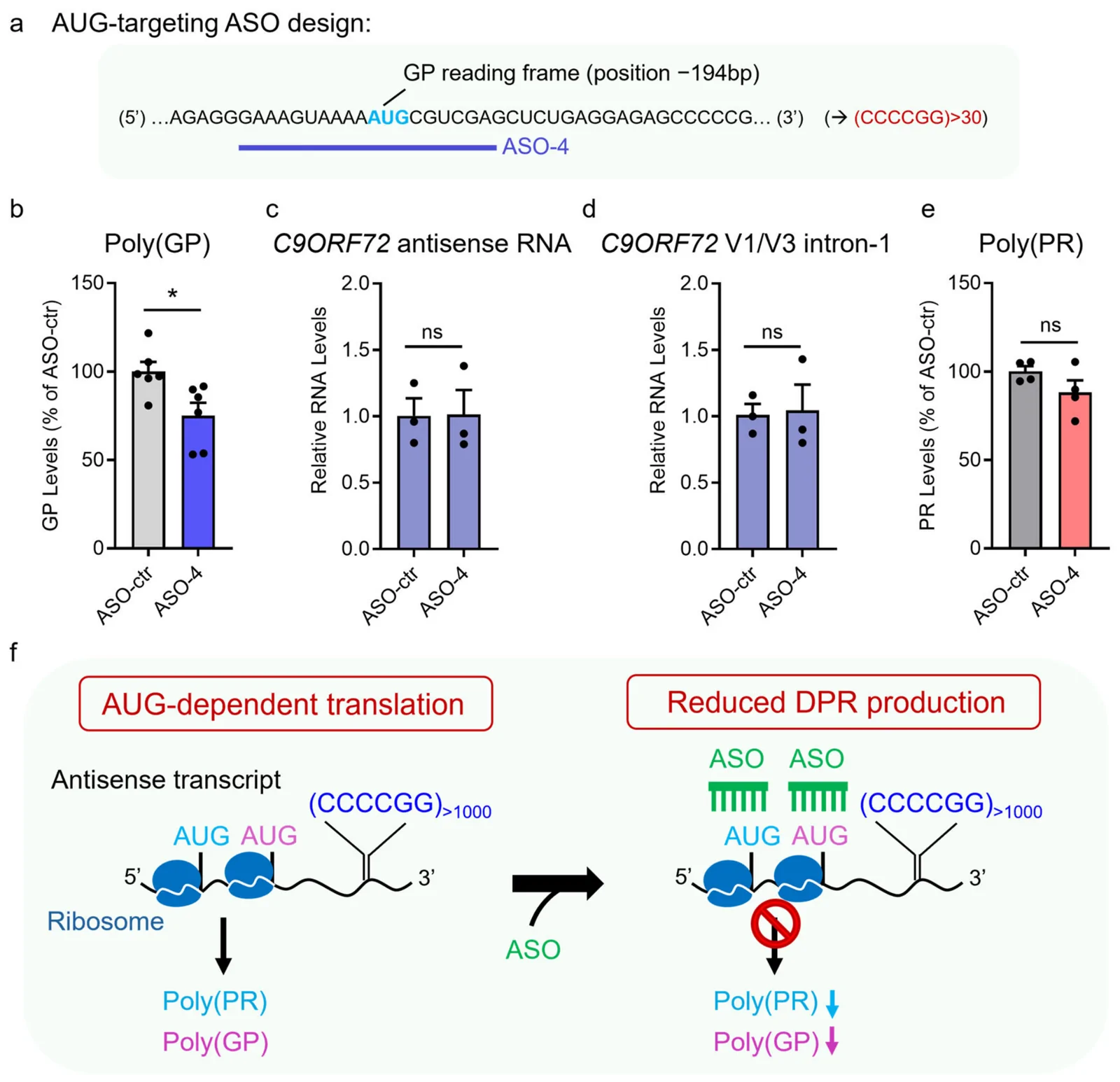
An antisense oligonucleotide (ASO) that sterically blocks an AUG start codon in the antisense repeat RNA suppresses poly(GP) protein synthesis. (**a**) Schematic of the steric-blocking ASO design targeting the AUG start codon located 194 bp upstream of the antisense repeat in the poly(GP) reading frame. (**b**) Poly(GP) protein levels in *C9ORF72* lymphoblastoid cell line-1 (LCL-1) following treatment with steric-blocking ASO-4 for 10 days. (**c**,**d**) Relative expression levels of *C9ORF72* antisense RNA and sense V1/V3 intron-1 RNA in *C9ORF72* LCL-1 cells treated with steric-blocking ASO-4 for 10 days, as determined by quantitative PCR. (**e**) Poly(PR) protein levels in *C9ORF72* LCL-1 cells following treatment with steric-blocking ASO-4 for 10 days. Values represent the mean ± s.e.m. from three to six independent cultures. * *p* < 0.05 (Student’s *t*-test); ns, not significant. (**f**) Schematic summary of the findings from the ASO experiments: AUG-dependent translation of antisense repeat RNA generates DPR proteins and is targetable by ASOs.

Following the successful use of ASOs targeting the AUG region in the poly(PR) reading frame, we designed an additional ASO (ASO-4) targeting the AUG codon located 194 bp upstream of the antisense repeats in the poly(GP) reading frame, which was previously identified as a major site of poly(GP) translation initiation in a reporter system [14]. Treatment of LCL-1 cells with ASO-4 significantly reduced poly(GP) protein levels by approximately 25% without affecting antisense RNA, sense intron-1 RNA or poly(PR) levels (Figure 5a–e). This modest reduction in poly(GP) levels is consistent with previous findings demonstrating that poly(GP) translation can also initiate from two alternative AUG codons within the same reading frame [22]. Together, these findings provide independent support for the functional importance of this AUG-containing region in poly(GP) production and are consistent with our CRISPR-Cas9 results.

Collectively, our findings establish that AUG-containing sequences upstream of the antisense repeat are critical determinants of poly(PR) and poly(GP) production, supporting a model in which antisense repeat RNA is translated predominantly through an AUG-dependent mechanism. Our ASO studies further demonstrate that these AUG-containing regions are amenable to therapeutic targeting and can be leveraged to reduce endogenous DPR production in *C9ORF72* carrier cells (Figure 5f).

## 4. Discussion

The mechanisms by which DPR proteins are synthesized from RNAs harboring the *C9ORF72* hexanucleotide repeat expansion remain incompletely understood. Multiple translation mechanisms likely contribute to the production of these proteins. Our findings highlight the critical role of sequences located upstream of the repeat expansion in regulating the synthesis of poly(GP) and poly(PR) in MNs and LCLs derived from *C9ORF72* repeat expansion carriers.

Using CRISPR-Cas9 genome editing and steric-blocking ASOs, we examined the contribution of AUG-containing sequences within the antisense repeat transcript to endogenous poly(GP) and poly(PR) production. Deletion of an AUG-containing region upstream of the antisense repeats markedly reduced poly(GP) levels without altering antisense RNA abundance, demonstrating that this sequence is required for efficient poly(GP) production. Analysis of transcript processing further revealed that unspliced sense transcripts containing the repeat expansion persist despite altered splicing, suggesting that these transcripts may serve as templates for poly(GA) and poly(GR) synthesis in human MNs. In addition, steric-blocking ASOs targeting AUG-containing sequences in the poly(PR) and poly(GP) reading frames significantly reduced poly(PR), and to a lesser extent, poly(GP) levels without affecting repeat RNA abundance, supporting a role for AUG-dependent translation of antisense repeat RNA. Collectively, these findings indicate that poly(GP) and poly(PR) production depends on AUG-containing sequences upstream of the antisense repeat and support a model in which these DPR proteins are generated through AUG-dependent translation initiation.

In the poly(GP) reading frame, three putative AUG initiation codons are located 212, 194, and 113 nucleotides upstream of the repeats and are not interrupted by in-frame stop codons. Any or all of these AUGs could therefore serve as translation initiation sites for poly(GP) synthesis. Boivin et al. [14] used mass spectrometry to analyze poly(GP) produced from antisense constructs and demonstrated that the AUG codon located 194 nucleotides upstream of the C4G2 repeats functions as the primary translation initiation site. Deletion of the antisense sequence containing this AUG abolished poly(GP) production [14]. Consistent with this finding, Sonobe et al. [22] demonstrated using a luciferase reporter system that although the AUG at position −194 is the preferred initiation codon, the AUGs at positions −212 and −113 can serve as alternative initiation sites when the −194 AUG is mutated [22]. Our data, obtained in the endogenous context of *C9ORF72* carrier cells, indicate that blocking the sequence containing the AUG at position −194 with an ASO is insufficient to completely abolish poly(GP) production, consistent with the existence of alternative AUG-dependent initiation sites [14,22]. Together, these observations suggest that effective suppression of poly(GP) production from antisense repeat RNA may require inhibition of translation initiation from all three AUG codons.

An important insight from this work is the clarification of the relative contributions of sense and antisense repeat RNAs to endogenous poly(GP) production. Our deletion studies indicate that antisense repeat RNA is the predominant source of poly(GP) in *C9ORF72*-derived MNs. This conclusion is supported by a study showing that CRISPR-Cas9-mediated excision of exon-1a in *C9ORF72* MNs abolished sense-derived poly(GA) production while having little effect on poly(GP) levels [34]. In that study, antisense RNA expression was largely preserved despite elimination of sense RNA, suggesting that poly(GP) is primarily generated from the antisense transcript [34]. Our findings, together with those of Sachdev et al. [34], are further supported with analyses of postmortem tissue from *C9ORF72* expansion carriers indicating that approximately 80% of poly(GP) originates from antisense repeat RNA [23]. However, these observations contrast with earlier reports showing that ASOs targeting sense *C9ORF72* RNA substantially reduce poly(GP) levels in cultured cells and in the cerebrospinal fluid of expansion carriers [24,25], indicating that poly(GP) can also be produced from the sense RNA despite the presence of a termination codon in frame with poly(GP). A possible reconciliation of these findings comes from a recent study demonstrating that a fraction of sense-derived poly(GP) may arise from a chimeric protein generated through translation initiation at a CUG codon in the poly(GA) reading frame [35]. Collectively, these data indicate that poly(GP) production is driven predominantly by antisense repeat RNA, with a smaller component potentially arising from alternative translation of sense transcripts.

While poly(GP) is considered one of the most soluble and least toxic DPR proteins, poly(PR) has emerged as one of the most toxic species, disrupting nucleolar function, ribosomal biogenesis, RNA metabolism, nucleocytoplasmic transport, stress granule dynamics, and protein homeostasis, ultimately leading to MN dysfunction and degeneration [26,27,28,29,36,37,38,39,40,41,42,43]. Defining how poly(PR) is produced is therefore essential for identifying therapeutic intervention points and for developing therapies capable of selectively suppressing this highly toxic DPR while minimizing effects on normal C9ORF72 function. Our findings indicate that the AUG initiation codon located 273 nucleotides upstream of the repeat expansion in the poly(PR) reading frame is required for efficient poly(PR) synthesis and may therefore represent a potential therapeutic target for reducing poly(PR) production. Several therapeutic strategies are currently being developed for *C9ORF72*-associated ALS/FTD, including antibody-based therapies, adeno-associated virus (AAV)-mediated gene delivery, and ASOs [44,45]. However, antibody therapies are limited by poor penetration of the blood–brain barrier [46,47], while AAV-mediated approaches face challenges including restricted packaging capacity, pre-existing and treatment-induced immune responses that limit repeat dosing, and difficulties achieving widespread CNS delivery [48,49,50,51]. In contrast, the clinical success of the ASO tofersen for SOD1-associated ALS has established ASOs as a promising therapeutic modality for genetically defined forms of ALS [52]. Realizing this potential for *C9ORF72* ALS/FTD, however, requires a detailed understanding of the molecular mechanisms governing DPR production to rationally design ASOs that selectively suppress toxic DPR synthesis while preserving normal *C9ORF72* expression. Together, our findings support the feasibility of an ASO-based strategy to reduce poly(PR) synthesis in cells derived from *C9ORF72* expansion carriers.

A recent ASO clinical trial targeting sense repeat RNA did not achieve the desired clinical outcomes in individuals with *C9ORF72*-associated ALS/FTD [53]. However, it remains unclear whether this outcome resulted from insufficient target engagement, suboptimal timing of therapeutic intervention, or a substantial contribution of antisense repeat-mediated pathologies, including poly(PR)-associated toxicity, to disease progression [53,54]. Our findings further support the possibility that targeting antisense repeat RNA or its translation machinery may represent a complementary therapeutic strategy for reducing pathogenic DPR production in *C9ORF72*-associated disease.

An important implication of this work is that DPR proteins contain unique N-terminal and C-terminal sequences in addition to the core repeat amino acid region [23]. Depending on which AUG codon is used for translation initiation, poly(GP) may contain N-terminal extensions of 72, 66, or 39 amino acids, whereas poly(PR) may contain a 91-amino-acid N-terminal extension. This observation is important because the amino acid composition of these terminal sequences could influence protein structure, subcellular localization, molecular interactions, and ultimately toxicity. Consistent with this possibility, inclusion of the native C-terminal sequence has been shown to reduce toxicity and alter the subcellular localization of DPR proteins [55]. Future studies investigating DPR-mediated toxicity should therefore incorporate native N-terminal and C-terminal sequences to ensure that experimental findings accurately reflect the endogenous disease context.

## 5. Conclusions

Our findings identify AUG-dependent translation initiation as a key mechanism underlying the synthesis of poly(GP) and poly(PR) from antisense *C9ORF72* repeat RNA. By demonstrating that AUG-containing sequences upstream of the repeat expansion are required for efficient DPR protein production, and that steric-blocking ASOs can selectively reduce poly(PR) synthesis without altering repeat RNA abundance, this work provides new insights into the molecular mechanisms regulating DPR synthesis. As therapeutic strategies for *C9ORF72*-associated disease continue to advance, a detailed understanding of the mechanisms controlling DPR translation will be critical for developing targeted interventions that reduce pathogenic DPR accumulation while preserving physiological C9ORF72 expression and function.

## Data Availability

The original contributions presented in this study are included in the article/Supplementary Material. Further inquiries can be directed to the corresponding author(s).

## Supplementary Materials

The following supporting information can be downloaded at: https://www.mdpi.com/article/10.3390/cells15181701/s1, Figure S1: Confirmation of the PCR products corresponding to the data presented in Figure 3. PCR products were amplified from cDNA using Taq polymerase to verify each primer set, separated on a 2\% agarose gel, and visualized with SYBR Safe to confirm the expected amplicon sizes. (a) Agarose gel image for *C9ORF72* variant 1 (V1). (b) Agarose gel image for *C9ORF72* variant 2 (V2). (c) Agarose gel image for *C9ORF72* variant 3 (V3). (d) Agarose gel image for the *C9ORF72* V1/V3 intron-1 amplicon.
