# Supplementary material for: AUG-Dependent Translation of Antisense Repeat Transcripts Contributes to Dipeptide Repeat Protein Production in C9ORF72 Expansion Carriers
Source: Cells. 2026 Sep 19;15(18):1701. doi: 10.3390/cells15181701 (PMC13605190; doi:10.3390/cells15181701)
Supplement: Supplementary file 1 [file cells-15-01701-s001.zip › cells-4539156-supplementary.pdf]

Supplementary Figure

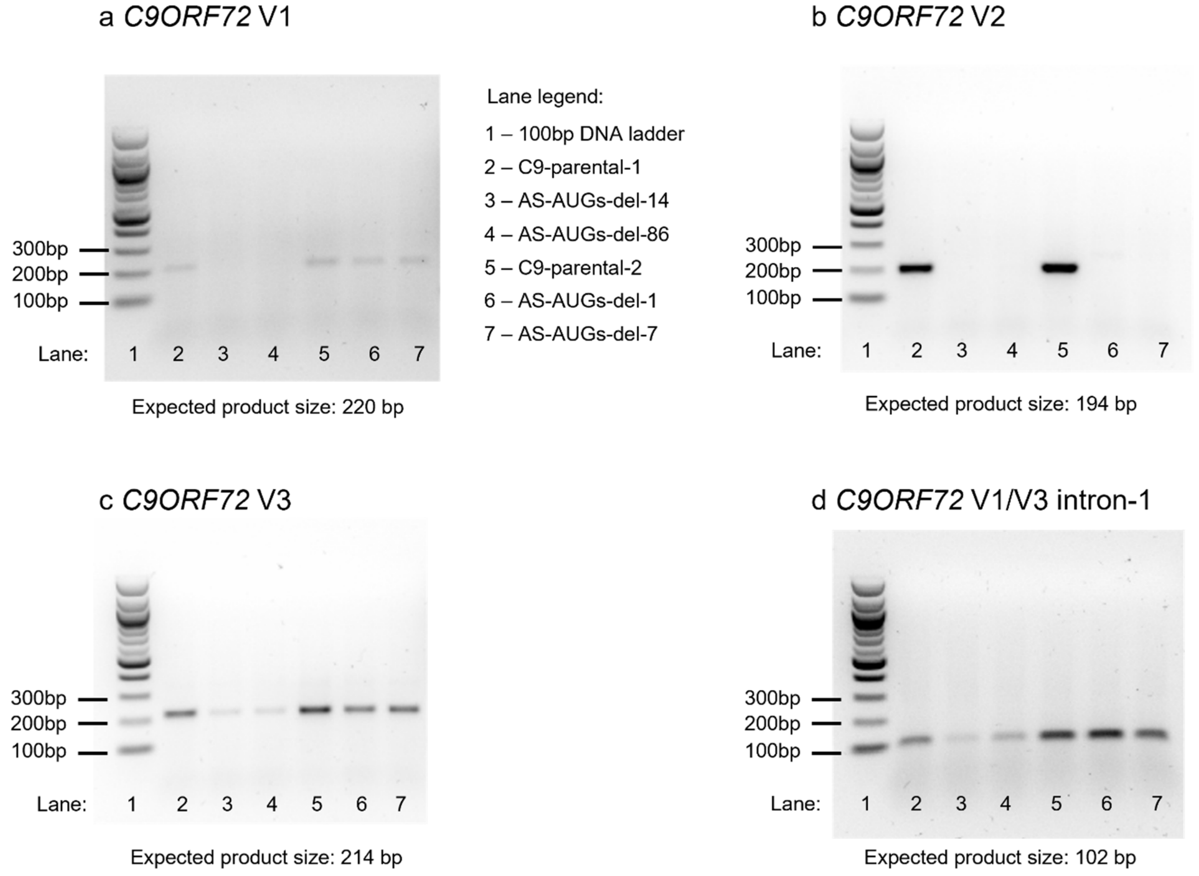

Supplementary Figure S1. Confirmation of the PCR products corresponding to the data presented in Figure 3. PCR products were amplified from cDNA using Taq polymerase to verify each primer set, separated on a 2% agarose gel, and visualized with SYBR Safe to confirm the expected amplicon sizes. (**a**) Agarose gel image for *C9ORF72* variant 1 (V1). (**b**) Agarose gel image for *C9ORF72* variant 2 (V2). (**c**) Agarose gel image for *C9ORF72* variant 3 (V3). (**d**) Agarose gel image for the *C9ORF72* V1/V3 intron-1 amplicon.
